# The phenotypic and genetic features of arrhythmogenic cardiomyopathy in the pediatric population

**DOI:** 10.3389/fcvm.2023.1216976

**Published:** 2023-09-15

**Authors:** Olga Kofeynikova, Daria Alekseeva, Tatiana Vershinina, Svetlana Fetisova, Olga Peregudina, Tatiana Kovalchuk, Elena Yakovleva, Polina Sokolnikova, Alexandra Klyushina, Kseniia Chueva, Anna Kostareva, Tatiana Pervunina, Elena Vasichkina

**Affiliations:** ^1^ World-Class Research Centre for Personalized Medicine, Almazov National Medical Research Centre, Saint Petersburg, Russia; ^2^ Department of Pediatric Cardiology, Almazov National Medical Research Centre, Saint Petersburg, Russia; ^3^ Institute of Molecular Biology and Genetics, Almazov National Medical Research Centre, Saint Petersburg, Russia; ^4^ Department of Women’s and Children’s Health and Center for Molecular Medicine, Karolinska Institutet (KI), Solna, Sweden; ^5^ Institute of Perinatology and Pediatrics, Almazov National Medical Research Centre, Saint Petersburg, Russia

**Keywords:** arrhythmogenic cardiomyopathy, children, sudden cardiac death, ventricular arrhythmia, task force criteria, Padua criteria

## Abstract

**Introduction:**

The present study aimed to describe the phenotypic features and genetic spectrum of arrhythmogenic cardiomyopathy (ACM) presented in childhood and test the validity of different diagnostic approaches using Task Force Criteria 2010 (TFC) and recently proposed Padua criteria.

**Patients and methods:**

Thirteen patients (mean age at diagnosis 13.6 ± 3.7 years) were enrolled using “definite” or “borderline” diagnostic criteria of ACM according to the TFC 2010 and the Padua criteria in patients <18 years old. Clinical data, including family history, 12-lead electrocardiogram (ECG), signal-averaged ECG, 24-h Holter monitoring, imaging techniques, genetic testing, and other relevant information, were collected.

**Results:**

All patients were classified into three variants: ACM of right ventricle (ACM-RV; *n* = 6, 46.1%), biventricular ACM (ACM-BV; *n* = 3, 23.1%), and ACM of left ventricle (ACM-LV; *n* = 4, 30.8%). The most common symptoms at presentations were syncope (*n* = 6; 46.1%) and palpitations (*n* = 5; 38.5%). All patients had more than 500 premature ventricular contractions per day. Ventricular tachycardia was reported in 10 patients (76.9%), and right ventricular dilatation was registered in 8 patients (61.5%). An implantable cardiac defibrillator was implanted in 61.5% of cases, and three patients with biventricular involvement underwent heart transplantation. Desmosomal mutations were identified in 8 children (53.8%), including four patients with *PKP2* variants, two with *DSP* variants, one with *DSG2* variant, and one with *JUP*. Four patients carried compound heterozygous variants in desmosomal genes associated with left ventricular involvement.

**Conclusion:**

Arrhythmias and structural heart disease, such as chamber dilatation, should raise suspicion of different ACM phenotypes. Diagnosis of ACM might be difficult in pediatric patients, especially for ACM-LV and ACM-BV forms. Our study confirmed that using “Padua criteria” in combination with genetic testing improves the diagnostic accuracy of ACM in children.

## 1. Introduction

Arrhythmogenic cardiomyopathy (ACM) (PS107970) is a genetic cardiac disorder characterized by progressive fibro-fatty replacement of the ventricular myocardium. The disease's natural causes include the gradual progression of ventricular arrhythmias (VA), right (RV) or, rarely, left ventricular (LV) dilation, and a decrease in systolic heart function with a high risk of sudden cardiac death (SCD) (1, 2). Initially, the disease was described as predominantly involving the right ventricle (ACM-RV). However, over the years, the definition of ACM has been extended, and currently, the disease is classified into three subtypes: ACM-RV, ACM-LV, and ACM-BV (3).

Diagnosing ACM can be challenging and is typically based on a combination of criteria, including a detailed family history, electrocardiogram (ECG), Holter monitoring (HM), echocardiography (echo), magnetic resonance imaging (CMR), endomyocardial biopsy (EMB), and genetic screening (1, 4). These clinical tests were summarized in a criterial-based diagnostic algorithm known as the revised Task Force Criteria (TFC) (5). However, the 2010 TFC are exclusively concerned with the right phenotypic manifestations of ACM, and as such, have several important limitations (1). To improve the diagnostic approach to ACM, the Padua criteria were proposed in 2020. These criteria include clinical examination on both ventricles (5–8).

Additionally, the Padua criteria take into account genotype information, which is especially important in the case of ACM-LV and ACM-BV. With current genotyping technologies, various groups of genes have been identified, predominantly associated with defined forms of ACM. For example, the most frequent ACM-RV is mainly associated with variants in desmosomal genes such as *PKP2*, *JUP*, *DSC2*, *DSG2*, *DSP*, and *SCN5A* (4–6). On the other hand, patients with ACM-LV or biventricular phenotype often harbor pathogenic variants in *LMNA*, *DSP*, *FLNC*, *TMEM43*, and rarely other genes such as *LDB3*, *DES*, *ACTN2*, *BAG3*, *PLN*, *TTN*, *NKX2-5*, *RBM20*, *SCN5A*, *KCNQ1*, *KCNH2*, *TRPM4*, *LEMD2*, *ILK* and mitochondrial DNA mutations (4–6, 9).

Although ACM mainly manifests in adulthood, it can be very aggressive in children, albeit very rarely. Several recently published extensive meta-analysis and clinical summaries regarding ACM in pediatric patients have confirmed this fact (10–12). However, data on the pediatric phenotype of ACM and its association with genetic background is still limited. Defining the phenotypic features of pediatric ACM in connection to the genetic background is crucial for clinical prognosis and risk stratification, genetic counseling, and decisions on device implantation. Therefore, the aim of the current study is to describe the phenotypic features and genetic spectrum of ACM presented in childhood and to test the validity of different diagnostic approaches using TFC 2010 and recently proposed Padua criteria in pediatric patients with ACM.

## 2. Materials and methods

### 2.1. Patient cohort

The study cohort consisted of pediatric ACM patients who were admitted to the Almazov National Medical Research Center between 2010 and 2022. Patients were included in the study if they had either a “definite” or “borderline” ACM diagnosis before the age of 18 years, as per revised TFC 2010 criteria and the new Padua criteria, with subdivision to ACM-RV, ACM-LV, or ACM-BV (7, 8). The clinical data analyzed included physical examination, laboratory tests such as electrolytes and enzymes, 12-lead ECG, signal-averaged ECG, HM, cardiac magnetic resonance (CMR, performed in 9 patients) and echocardiography. Echocardiography criteria of RV/RV outflow tract (RVOT)/LV dilatation and/or dysfunction were as follows: following: RV dilatation >2 *z*-score (13), FAC <35%, PLAX RVOT/BSA ≥16 mm/m^2^, PSAX RVOT/BSA ≥18 mm/m^2^, LV dilatation > *z*-score 2 (14), ejection fraction LV <50% and wall motion abnormalities of LV or RV. LV dilatation and/or dysfunction by CRM was defined by identification of the following: end-diastolic volume ≥120 ml/m^2^, EF <50%; RV—RVEF <40%, end-diastolic volume ≥120 ml/m^2^ in male and ≥110 ml/m^2^ in female subjects and wall motion abnormalities. Histopathological examination was performed in 5 patients (patients 3, 4, 7, 9, 10): the samples were stained with hematoxylin and eosin and van Gieson stain. We performed immunohistochemical analysis with antibodies to CD3, CD68, HLA-DR to exclude myocarditis. Morphometric study of the residual area of cardiomyocytes and calculation of cell infiltrate were carried out using an image analyzer Image Scope Color M (Russia).

The study was performed in accordance with the Declaration of Helsinki, and approval was obtained from the local ethical committee of Almazov National Medical Research Center (15). Written informed consent was obtained from the parents of the minors before the investigation.

### 2.2. Genetic testing

Genotyping was performed using a target sequencing panel that included the 172 cardiomyopathy-related genes as previously described (16). In brief, target panel of 172 cardiomyopathy-associated genes was designed and processed with the SureSelect Target Enrichment System (Agilent; Waldbronn, Germany) with an Illumina MiSeq instrument (see Supplementary Material), probeset regions were extended by 100 bp in both directions to capture variants in non-coding areas during downstream analysis.

Alignment, data processing, and variant calling were performed according to GATK BestPractice recommendations using hg38 human genome reference and following tools were used: nextflow v22.10.2, python v3.10.6, fastp v0.23.2, fastqc v0.11.9, bwa v0.7.17, bcftools v1.16, samtools v1.16.1, mosdepth v0.3.3, gatk4 v4.3.0.0, vcftools v0.1.16. For variant calling HaplotypeCaller v4.3.0.0 and Deepvariant v1.4.0 were used. Filtered variants were annotated with Variant Effect Predictor (VEP) v.104 using frequency data from gnomAD, TOPMed and ExAC databases. Additionally, CADD v1.6 and dbNSFP v4.2 databases were used to assign pathogenicity prediction scores to variants. Variant annotation was performed using ANNOVAR (Philadelphia, PA, USA). The variants were filtered based on their functional consequences. To assess the influence of variants on splicing, we utilized Sequence Ontology (SO) classifications provided by VEP, along with precomputed SpliceAI scores from Gencode v24 by Illumina and dbscSNV v1.1 scores from dbscSNV. These resources were integrated as plugins within VEP. New ClinVar records were also incorporated during analysis. Before undergoing manual curation, variants were selected based on the following criteria: genotype quality >20, coverage depth >5 for SNPs and >10 for InDels, AF in gnomAD <0.01, CADD score >20, non-Benign classification in ClinVar, protein sequence altering or splicing altering consequences. Finally, bidirectional Sanger sequencing was performed to validate all reported variants. Genetic variants were classified according to the 2015 American College of Medical Genetics and Genomics criteria (ACMG) (17).

## 3. Results

### 3.1. Patient cohort

The study cohort consisted of 13 children with confirmed ACM, according to the TFC 2010 criteria and Padua criteria, who were admitted to the Almazov National Medical Research Center. The cohort comprised: 5 females (38.5%) and 8 males (61.5%) (Table 1), with a mean age at diagnosis of 13.6 ± 3.7 years. Twelve patients had a “definite” diagnosis according to both TFC 2010 criteria and Padua criteria, while one patient had a “borderline” diagnosis (Table 2). All patients were classified into three variants: ACM-RV (6 patients, 46.1%), ACM-BV (3 patients, 23.1%), and ACM-LV (4 patients, 30.8%) (Table 1).

**Table 1:** Genetic and phenotypic characteristics of patients.

	Pt.1	Pt.2	Pt.3	Pt.4	Pt.5	Pt.6	Pt.7	Pt.8	Pt.9	Pt.10	Pt.11	Pt.12	Pt.13
Gender	m	m	m	m	m	m	m	f	f	f	f	f	m
Phenotype	RV	RV	RV	RV	RV	LV	LV	LV	BV	BV	BV	BV	LV
Genotype	*RYR2* LP	*PKP2/PKP2* LP/LP	*PKP2* LP	*SYNE1* VUS	*DSG2* P	*DSP/DSP* P/P	*FLNC* VUS	*JUP* VUS	*MYH7/FKTN/ANK2* P/VUS/VUS	*PKP2/PKP2* LP/VUS	*PKP2/PKP2* LP/P	*DSP* VUS	*SCN5A/ANK2* P/VUS
Outcome	-	ICD	ICD HT	-	ICD	ICD	ICD	-	ICD HT	ICD HT	ICD	-	-
Age at diagnosis (years)	12	17	15	16	13	5	17	17	16	13	8	12	16
Age at first symptoms	11	15	14	12	13	4	14	17	12	12	4	12	16
Family histoty													
SDD	-	Maternal grandfather	Father	-	-	-	Father	-	-	-	-	-	-
Phenotype of parents	n/a	-	n/a	n/a	n/a	-	n/a	n/a	-	-	-	n/a	n/a
Genotype of parents	n/a	Mother PKP2 Father PKP2	n/a	n/a	n/a	n/a	n/a	n/a	n/a	Mother PKP2	Mother PKP2 Father PKP2	n/a	n/a
First symptoms													
Syncope	+	−	+	−	−	−	+	+	−	−	−	+	−
Palpitations	−	+	−	−	−	−	−	−	+	+	+	−	+
Chest pain	−	−	−	+	+	−	−	+	+	+	−	−	−
“Hot phase”	−	−	−	+	+	−	−	+	+	+	−	−	−
Functional class of heart failure (at the first admission)	II	-	II	II	II	II	II	-	II	II	II	II	-
ECG anomalies													
Negantive T wave in V1–V3	−	−	−	−	−	−	−	−	+	−	−	−	−
Epsilon wave	−	−	−	−	−	−	−	−	−	−	−	−	−
Late potentials	+	+	−	+	+	−	−	+	+	−	+	+	+
>500 PVCs/24 h	+	+	+	+	+	+	+	+	+	+	+	+	+
VT	+	+	+	−	+	+	−	−	+	+	+	+	+
VT polymorphic	+	−	+	−	−	+	−	−	−	+	+	−	+
Echocardiographic findings													
RV dilatation	+	+	+	−	+	−	−	−	+	+	+	+	−
LV dilatation	−	−	−	+	−	+	+	+	+	+	+	−	+
Aneurysm of RV and LV	−	−	−	−	−	−	−	−	+	+	−	−	−
CMR features													
RV dysfunction	+	−	n/a	−	+	−	−	−	+ Aneurysm	n/a	+ Aneurysm	n/a	n/a
LV dysfuction	−	−	n/a	−	−	+	+	−	+	n/a	−	n/a	n/a
Fibrosis (LGE)	+	−	n/a	−	+	+	+	−	+	n/a	+	n/a	n/a

BV, biventricular; f, female; HT, heart transplantation; ICD, implantable cardioverter-defibrillator; LV, left ventricle; CMR, magnetic resonance imaging; PVCs, premature ventricular complexes; RV, right ventricle; SDD, sudden cardiac death; VT, ventricular tachycardia; n/a, not available or not performed; P, pathogenic; LP, likely pathogenic; VUS, variant of unknown significance.

**Table 2:** ACM criteria (comparison of the TFC 2010 and the Padua criteria).

Patient	Task force criteria 2010							The Padua criteria						
	Structural	Tissue	Repolarization	Depolarization	Arrhythmias	Family history and Genetics	Diagnosis	ECG	Arrhythmia	Imaging	CMR	Biopsy	Genetics	Diagnosis
Pt.1	M (CMR)	–	–	m	m	–	Definitive RV	–	m	M	M	–	–	Definitive RV
Pt.2	–	–	–	m	m, m	M	Definitive RV	–	m	–	–	–	M	Borderline RV
Pt.3	M (Echo)	–	–	–	m	M	Definitive RV	–	m	M	–	–	M	Definitive RV
Pt.4	–	M	m	m	m	–	Definitive RV	m	m	–	–	M	–	Definitive RV
Pt.5	M (CMR)	–	–	m	m, m	M	Definitive RV	–	m	M	M	–	M	Definitive RV
Pt.6	–	–	–	–	–	M	Possible RV	–	m	m	M	–	M	Definitive LV
Pt.7	–	–	–	–	m	M	Possible RV	–	m	M	M	–	M	Definitive LV
Pt.8	–	–	–	m	–	M	Possible RV	m	m	m	–	–	M	Definitive LV
Pt.9	M	M	M	m	m	–	Definitive RV	m	m	M	M	M	–	Definitive BV
Pt.10	M	M	–	–	m	M	Definitive RV	–	m	M	–	M	M	Definitive BV
Pt.11	M	–	–	m	m	M	Definitive RV	–	m	M	M	–	M	Definitive BV
Pt.12	m	–	–	m	m, m	M	Definite RV	–	m	m	–	–	M	Definitive RV
Pt.13	–	–	–	m	–	M	Possible RV	–	m	M	–	–	M	Definitive LV

BV, biventricular; echo, echocardiography; LV, left ventricle; M, major criteria; m, minor criteria; CMR, magnetic resonance imaging; RV, right ventricle.

Table 1 shows the clinical characteristics of the patients. The mean age of symptom onset was 12 ± 3.95 years (range 4–17), with syncope reported in 6 patients (46.1%), palpitations in 5 patients (38.5%), and chest pain in 5 patient (38.5%). Only 1 patient (7.7%) remained asymptomatic at the time of diagnosis and was examined due to premature ventricular contractions (PVCs) at 12-lead ECG baseline screening during a routine cardiology visit. Importantly, a “hot phase” of clinical presentation with chest pain episodes and high troponin I and/or CPK values was documented in 5 children (38.5%) after thorough exclusion of myocarditis according to MRI, cardiac biopsy and serum data. Heart failure was present in all pediatric patients at the time of the first admission, with 10 patients (76.9%) classified as NYHA functional class II.

All patients had available ECG and HM for analysis. ECG abnormalities were evident in all cases (100%). More than 500 PVCs per day were documented in all pediatric patients, and ventricular tachycardia (VT) was reported in 10 patients (76.9%), including 6 cases (46.1%) of polymorphic VT. The origin of monomorphic VT in the remaining 3 (23.1%) patients was in the RVOT with left bundle branch block (LBBB) morphology with inferior axis, one patient had monomorphic VT with left bundle branch block (LBBB) morphology with superior axis (7.7%), all of which met the major criteria of TFC 2010. Negative T waves in the right chest leads (V1–V2, V1–V4) were observed in 2 adolescents (15.4%) at the age of 16, but only one of them had it as the main criterion. None of the 13 patients had a defined and clear visualization of the epsilon wave while late potentials were seen in 9 patients (69.2%), no patient presented with low ECG including those with ACM-BV or ACM-LV. Regardless of the ACM phenotype, VA was the most prevalent symptom leading to the diagnosis of ACM. Children diagnosed with ACM-BV or ACM-LV had a poorer prognosis due to severe heart failure (Table 1).

### 3.2. Imaging and visualization

Echocardiographic abnormalities were detected in all 13 cases after the initial clinical presentation. Dilatation of the RV was registered in 8 patients (61.5%), 6 of whom had dilatation of the RVOT, LV dilatation was observed in 8 patients (61.5%). Biventricular involvement was documented in 3 out of 13 pediatric cases (23.1%), and aneurysms of the LV and RV were described in only 2 patients (15.4%). CMR was available for only 9 children and confirmed the diagnosis in 6 of them. Regional RV wall motion abnormalities, such as dyskinesia or akinesia, were noted in 4 patients (30.7%), two of whom had RV aneurysm. Late gadolinium enhancement (LGE) evidence of fibrosis was registered in 6 children (46.1%), including 2 cases (15.4%) where it was solely in the RV, 2 cases (15.4%) where it was solely in the LV, and 2 cases (15.4%) where it was present in both ventricles. Only 3 patients (23.1%) presented with LV regional wall motion abnormalities (hypo/akinesia), according to CMR data.

### 3.3. Histopathological examination

Histopathological examination was performed in 5 patients (Table 3); of those, 2 patients underwent a diagnostic EMB during radiofrequency ablation and 3 patients underwent histopathological examination of native heart. The morphological diagnosis of ACM was confirmed in all 5 patients. In all cases, the residual cardiomyocyte area was less than 40%. In 1 patient who had a biventricular phenotype, the residual cardiomyocyte area corresponded to 5%–10%. In 3 cases, fibro-fatty replacement were detected.

**Table 3:** Data of histopathological examination.

	Condition	Residual myocardium (%)	Fibrosis	Lipomatosis	Inflammation
Pt3	Native heart	37%	+	−	+
Pt4	Diagnostic EMB	38%	+	+	−
Pt7	Diagnostic EMB	-	+	−	−
Pt9	Native heart	5%–10%	+	+	+
Pt10	Native heart	<40%	+	+	+

EMB, endomyocardial biopsy.

### 3.4. Management of patients

All patients received antiarrhythmic therapy, including beta-blockers in 7 patients (53.8%), sotalol in 4 children (30.8%), amiodarone in 1 patient, and a combination of propafenone and sotalol in 1 patient. Almost half of the patients (7 of 13, 53.8%) required heart failure therapy, including diuretics and ACE inhibitors. Radiofrequency ablation of VT was attempted in 4 patients but was unsuccessful. Implantable cardioverter-defibrillators (ICDs) were implanted in 8 children (61.5%). Overall, 3 (23.1%) patients underwent cardiac transplantation before the age of 18 (at the age of 16 years), and one of them needed prolonged mechanical support prior to heart transplantation. All transplanted patients were followed in the outpatient department even after achieving 18 years of age and have remained stable until now.

### 3.5. Genetic testing

Genetic testing was performed in all 13 patients using a targeted new-generation sequencing approach with a broad spectrum of genes examined [Supplementary File, (13)]. This led to the identification of the genetic cause in all 13 patients. Desmosomal mutations were detected in 8 children (53.8%), including 4 patients with *PKP2* variants, 2 patients with *DSP* variants, 1 patient with *DSG2* variant, and 1 patient with *JUP* (Table 1). Three patients were compound heterozygous and carried two *PKP2* variants, and 1 patient carried two variants in *DSP* with the corresponding clinical features of Сarvajal syndrome. Genetic variants in non-desmosomal genes, such as *FLNC*, *MYH7*, *RYR2*, *SCN5A*, and *SYNE1*, were found in 5 patients (38%).

### 3.6. Family screening

**Figure 1:**
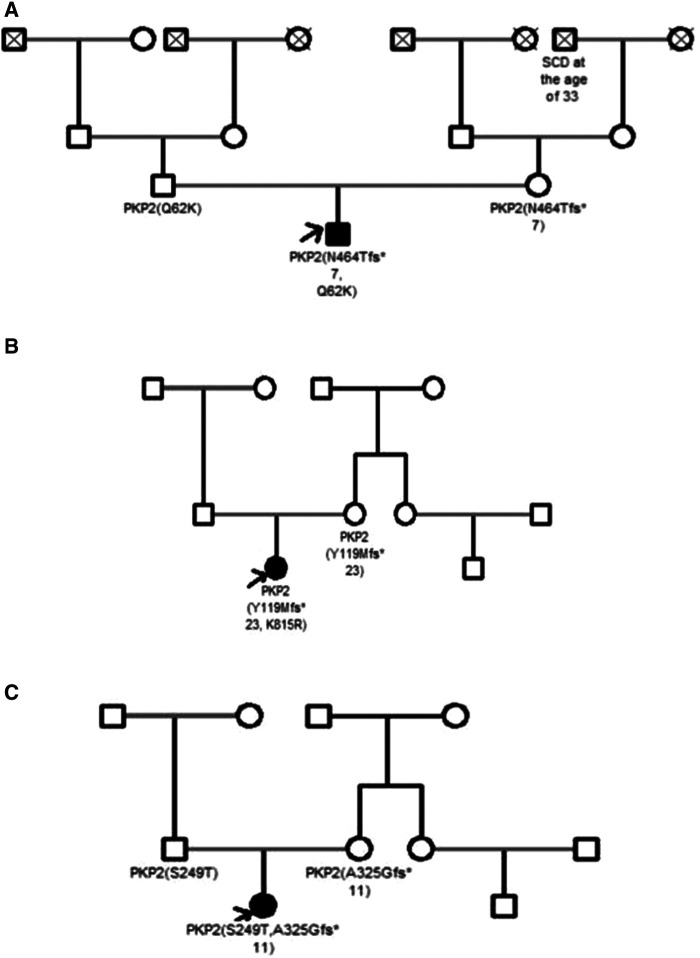
Pedigrees of the family **A** (patient 2), family **B** (patient 10) and family **C** (patient 11). ACM patients are shown with solid symbols and unaffected with white symbols, the arrow indicates the proband. Dead people are shown with a cross. SCD, sudden cardiac death.

None of the patients had a family history of ACM, but SCD was reported in three families (23.1%). Clinical cardiac screening was performed in 5 out of 13 (38.5%) families and all relatives (parents and siblings) were asymptomatic for the moment. The data of genetic screening were available only in three families with compound PKP2 variants detected in the probands (patient 2, 10 and 11). In all three cases one or two parents were carriers of single PKP2 variant, still, remained asymptomatic for the time of clinical investigation (Table 1, Figure 1).

## 4. Discussion

According to the 2019 HRS expert consensus document, ACM-RV is considered to be extremely rare in children under the age of 10 years. Therefore, diagnosing ACM in childhood is quite challenging (18, 19). It is still under debate to what extent the TFC 2010 and Padua criteria of ACM are sensitive and specific in the pediatric population, taking into account that many of their components, such as CMR and EMB, often cannot be performed in children. Meanwhile some criteria, such as T-wave inversion or epsilon wave, may not provide enough information. In this regard, comparing the specificity and sensitivity of these criteria, especially for various forms of ACM, remains an important clinical task in pediatric cardiology.

We used the TFC 2010 and Padua criteria for the diagnosis of ACM. In patients with ACM-RV, the application of TFC 2010 and Padua criteria yielded consistent results. Padua criteria confirmed the definitive diagnosis in 5 out of 6 patients with ACM-RV (patients 1, 2, 3, 5, and 12). Only in 1 patient (patient 4), the diagnosis of definite ACM-RV was slightly weakened but still confirmed as borderline. In addition, in the other 3 patients (patients 9, 10, and 11), the ACM-RV suggested by TFC 2010 was revised to ACM-BV after reassessment using Padua criteria. In addition to 4 patients (6, 7, 8, and 13) initially diagnosed with ACM-LV using only Padua criteria, a total of 7 patients received a non-ACM-RV diagnosis when solely applying Padua criteria. Considering the prevalence of non-ACM-RV in half of the pediatric patients in our group and its worse prognosis compared to ACM-RV, the implementation of Padua criteria in children becomes more favorable, leading to a more precise diagnosis and risk stratification. This is in line with recent data published by Cicenia et al. on a group of 21 children, which also confirmed the superior performance of Padua criteria in establishing ACM diagnosis in children (12).

Of note, despite the early and severe clinical course, none of our patients reported a family history of ACM, which is a well-known hereditary disease. Even when considering 3 cases of SCD in close relatives, this may correspond to only 23% of familial cases. This fact can potentially be explained by several reasons. First, compound heterozygosity forms suggest that at least one of the parents carries a pathogenic variant which can be non-penetrant or lead to the late manifestation. This phenomenon is widely described for ACM variant carriers (18–21). In this case, only a compound heterozygous combination of variants can lead to the early and severe manifestation being asymptomatic in parents with single variants (as in patients 6 and 10). Another explanation is the potential *de novo* status of the variants, the most likely mechanism in non-desmosomal gene variants (*LMNA*, *MYH7*, *FLNC*), leading to the ACM-BV. Thus, Klauke et al. confirmed the association between *de novo* status in *DES* and ACM through both clinical and functional characterization (22). Therefore, the inability to confirm the *de novo* or allelic status of the mutations due to the unavailability of parental DNA can be an important limitations of our study.

Riele et al. showed that clinical phenotype and ARVC course were similar between pediatric and adult cases, and ACM may be fully manifested in pediatric patients with VA, cardiac failure, and SCD (23). Our study confirmed the prevalence of arrhythmic symptoms in pediatric ACM since arrhythmogenic manifestations (VA, including VT) were reported in all children from our study group. However, only 1 patient had VT of LBBB with a superior axis, which met the major criteria according to the TFC 2010. All other patients initially had VT of different morphology, so the diagnosis of ACM was confirmed using other criteria. These results questioned the sensitivity of arrhythmic criteria in the pediatric population and suggested that any type of VT could be a sign of ACM in children. This notion was previously underlined by Shriprasad et al., providing a further argument for establishing modified ACM diagnostic criteria in children (24). In most cases, arrhythmia preceded structural changes in the heart. Therefore, establishing a diagnosis in time to work on SCD prevention is extremely important (10, 25–27). Our results showed that children with ACM-RV and ACM-BV forms had the most pronounced and difficult-to-treat VA; 8 out of 9 children in this group required ICD implantation. In this light, it is necessary to provide dynamic monitoring of children with cardiac arrhythmias with or without any structural myocardial pathology to establish ACM diagnosis on time.

One the most challenging criteria to apply in children when diagnosing ACM is depolarizing abnormalities, since inverted T waves is a common finding in this population (28). As expected, only 1 child in our cohort met the main criterion for repolarization, which was a negative T wave in V1, V2, V3, and V4 leads. Additionally, epsilon waves are extremely rare in pediatric patients (26, 29). To confirm this, none of our patients had a clear visualization of the epsilon wave, which questions its reliability as a diagnostic criterion in pediatric patients.

Undoubtedly, CMR is a highly valuable in diagnosing ACM, and its high specificity in children has been validated by several studies (10–13, 26). In our cohort, dilatation of the RV or LV was detected in all patients, and signs of fibrosis on CMR were major criteria in 6 out of 9 patients, in whom CMR was available. However, performing CMR in young children can often be challenging due to the need for anesthesia, and the risk of severe cardiac arrhythmia (30, 31). It is worth noting that, the commonly used CMR parameters of TFC 2010 have not yet been validated in pediatric patients (26). Similar limitations are associated with EMB performance. While EMB can be very informative in establishing the diagnosis of ACM, its utility is rather limited in children due to the high risk of complications (24).

Since children lack a gold standard for diagnosing ACM, genetic testing can play a key diagnostic role, especially in cases of ACM-BV and ACM-LV (8, 32). Similar to adult groups, mutations in one of the desmosomal genes were the most frequent genetic causes in our study group, with 8 out of 13 patients having such mutations (*PKP2*, *DSP*, *DSG2*, and *JUP*) (1, 3, 33, 34). Of note, in half of the cases caused by desmosomal gene mutations, compound heterozygosity was detected mainly in association with LV involvement (patients 6, 10, and 11). In contrast, most of the isolated desmosomal gene mutations (3 out of 4) were associated with ACM-RV (Patients 2, 5, and 12). Even considering the small number of patients studied, one can suggest that compound heterozygosity in desmosomal genes is associated with a poor prognosis and early involvement of the LV in pediatric patients with ACM (35). Non-desmosomal genes (*FLNC*, *RYR2*, *MYH7*, *SCN5A*, and *SYNE1*) were detected in more than one-third of the children in our group. While some of these genes are well-known to be associated with ACM (*SCN5A*), the causative role of others, such as, *SYNE1*, needs further verification.

The present study evaluates the usefulness of TFC 2010 and Padua criteria in diagnosing ACM in a cohort of 13 pediatric patients. Interestingly, in 2 patients diagnosed with ACM, pathogenic variants were identified in genes that are well-known to be associated with other types of inherited cardiac disorders, *MYH7*, *FLNC* and RYR2 (6, 36, 37). These molecular mechanisms can differ from those in desmosome-associated ACM, potentially, leading to different clinical approaches. Both patients met the TFC 2010, and one of them, who carried the *RYR2* variant, had one major CMR criterion of ACM. This highlights the clinical and genetic overlap between ACM and other inherited cardiac disorders, emphasizing the need for long-term close follow-up for a better understanding of such phenotypes (6).

Protonotario et al. reported that the sensitivity and specificity of the ACM-risk prediction model depend on the genotype with different factor weights depending on the causative gene (30). DeWitt et al. showed that ACM-RV is associated with a high proportion of PKP2 variants, while ACM-LV is associated with DSP (11). Although this study mainly includes desmosomal gene mutations, similar patterns, even to a greater extent, can be useful for ACM associated with non-desmosomal genes. Similar to other reports, we confirmed that ACM-LV and ACM-BV in children have some important clinical features. Thus, an earlier onset of the disease was noted in children with ACM-LV and ACM-BV. ACM-BV was the most severe and 2 out of 3 patients in the group required heart transplantation in childhood, mainly due to the rapid progression of biventricular heart failure. This notion was previously reported by Somprasong et al., who confirmed that the involvement of the LV in children is a stronger predictor of adverse outcomes, including the need for heart transplantation (38). Given the severity of ACM-LV- and ACM-BV and the difficulties in their accurate diagnosis, we emphasize the need for the proper use of Padua criteria in children, in combination with extensive genetic screening in order to establish this clinical form in a timely manner and to ensure the appropriate risk management, family screening, and genetic counseling.

## 5. Conclusion

Using a cohort of 13 pediatric patients diagnosed with ACM, we assessed the utility of TFC 2010 and Padua criteria. We demonstrated that VT of any morphology, in combination with chamber dilatation, should raise the suspicion of different ACM phenotypes. Age-related electrocardiographic features in children do not allow for the reliable use of the existing ECG diagnostic criteria for ACM in pediatric patients. The proportion of non-ACM-RV forms in pediatric patients is close to 50% and is associated with multiple gene mutations, LV involvement, and poor prognosis. Further analysis of larger pediatric cohorts with ACM due to different genetic backgrounds will allow for establishing more reliable genotype-phenotype correlations and creating new, more sensitive diagnostic criteria for ACM children with different phenotypes.

## 6. Study limitations

The study has some limitations that need to be acknowledged. First, it was a retrospective study that was limited to a small cohort of patients. Second, some clinical data were not available to all patients, for example, CMR was missed for 4 out of 13 patients, and EMB was not possible to perform in some of them. Another important limitation is the unavailability of parental DNA in most cases, which made establishing the *de novo* or inherited status of a genetic variant and its penetrance in parents impossible. This absence of detailed segregation analysis underlines the need for further research and functional studies for the new described variants and genes.

## Data Availability

The datasets presented in this study can be found in online repositories. The names of the repository/repositories and accession number(s) can be found in the article/**Supplementary Material**.
