# Supplementary material for: The phenotypic and genetic features of arrhythmogenic cardiomyopathy in the pediatric population
Source: Front Cardiovasc Med. 2023 Sep 15;10:1216976. doi: 10.3389/fcvm.2023.1216976 (PMC10541206; doi:10.3389/fcvm.2023.1216976)
Supplement: Supplementary file 1 [file Table1.docx]

***Supplementary Material***

**The phenotypic and genetic features of arrhythmogenic cardiomyopathy in the pediatric population.**

Olga Kofeynikova , Daria Alekseeva , Tatiana Vershinina, Svetlana Fetisova, Olga Peregudina, Tatiana Kovalchuk , Elena Yakovleva , Polina Sokolnikov , Alexandra Klyushina, Kseniia Chueva, Anna Kostarev , Tatiana Pervunina, Elena Vasichkina

*** Correspondence:Olga Kofeynikova kofeolyaa@gmail.com**

Supplementary Data

Comprehensive_Cardiac_Panel (Agilent; Waldbronn, Germany) Design ID:3176121 (810.587 kbp)

ABCC9, ACADVL, ACTA1, ACTC1, ACTN2, ACVR2B, AGK, AKAP9, ALPK3, ANK2, ANKRD1, ANO5, BAG3, BRAF, CACNA1C, CACNA2D1, CACNB2, CALM1, CALM2, CALM3, CALR3, CASQ2, CAV3, CBL, CDH2, CMYA5, CRELD1, CRYAB, CSRP3, CTNNA3, DES, DMD, DMPK, DNAAF1, DNAAF3, DPP6, DSC2, DSG2, DSP, DTNA, DYSF, EMD, EYA4, FHL1, FHL2, FHOD3, FKRP, FKTN, FLNA, FLNC, FXN, GAA, GATA4, GATA5, GATA6, GATAD1, GDF1, GJA5, GLA, GPD1L, HAND1, HCN4, HFE, HRAS, ILK, ISPD, JPH2, JUP, KCNA5, KCND3, KCNE1, KCNE2, KCNE3, KCNE5, KCNH2, KCNJ2, KCNJ5, KCNJ8, KCNQ1, KRAS, LAMA4, LAMP2, LDB3, LEFTY2, LMNA, LMOD3, LRRC10, LZTR1, MAP2K1, MAP2K2, MIB1, MMP21, MRAS, MYBPC3, MYBPHL, MYH6, MYH7, MYL2, MYL3, MYL4, MYLK2, MYOF, MYOM1, MYOT, MYOZ2, MYPN, NEBL, NEXN, NF1, NKX2-5, NKX2-6, NPPA, NRAS, NUP155, PDLIM3, PKD1L1, PKP2, PLEC, PLEKHM2, PLN, PPA2, PPP1CB, PRDM16, PRKAG2, PSEN1, PSEN2, PTPN11, RAF1, RANGRF, RBM20, RIT1, RRAS, RYR2, SALL4, SCN10A, SCN1B, SCN2B, SCN3B, SCN4B, SCN5A, SCNN1G, SDHA, SGCD, SHOC2, SLMAP, SNTA1, SOS1, SOS2, SPEG, SPRED1, SYNE1, SYNM, SYNPO2L, TAZ, TBX20, TBX5, TCAP, TECRL, TGFB3, TMEM43, TMPO, TNNC1, TNNI3, TNNI3K, TNNT2, TPM1, TRDN, TRPM4, TTN, TTR, VCL, ZIC3

# Supplementary Table 1

Genetic variants identified in ARVC paediatric cases,

| Patient | Gene | Variants | ACMG  Classification | CADD |
| --- | --- | --- | --- | --- |
| Patient 1 | RYR2 | Chr1:237806240,  NM_001035.3:c.14255C>T:p.**T4752I** | LP (PP3, PM1, PM2) | 26 |
| Patient 2 | PKP2 | Chr12:32841178-32841193,  NM_001005242.3:c.1391_1406del:p.**N464Tfs*7** | P (PVS1, PP5, PM2) | - |
|  | PKP2 | Chr12:32896548, rs199601548,  NM_001005242.3:c.184C>A:p.**Q62K** | VUS (BP1, PP3, PP4) | 43 |
| Patient 3 | PKP2 | Chr12:32841178-32841193, NM_001005242.3:c.1391_1406del:p.**N464Tfs*7** | P (PVS1, PP5, PM2) | - |
| Patient 4 | SYNE1 | Chr6:152330437, rs12213435,  NM_182961.4:c.14248G>T:p.**D4750Y** | VUS (PM2, BP1) | 21 |
| Patient 5 | DSG2 | Сhr18:31519867, rs121913006,NM_001943.5:c.146G>A:p.**R49H** | P (PP5, PP3, PM1, PM5, PM2) | 26 |
| Patient 6 | DSP | Chr6:7559251,  NM_004415.4:c.448C>T:p.**R150*** | P (PVS1, PP5, PM2) | 37 |
|  | DSP | Chr6:7580877-7580878, NM_004415.4:c.4687_4688del:p.**L1563Efs*63** | P (PVS1, PP5, PM2) | - |
| Patient 7 | FLNC | Chr7:128845999, rs768767784,  NM_001127487.2:c.3800G>A:p.**R1267Q** | VUS (PP3, PM2, BP1) | 32 |
| Patient 8 | JUP | Chr17:41756183, rs782475413,  NM_002230.4:c.2078A>G:p.**Y693C** | VUS (PM2, PP3) | 22 |
| Patient 9 | MYH7 | Сhr14:23424839, rs36211715, NM_000257.4:c.2609G>A:p.**R870H** | P (PP5, BM1, BM5, PP3, PM2) | 25 |
|  | FKTN | Chr9:105604282, rs143748939, NM_001079802.2:c.437G>A:p.**R146Q** | VUS (PP3, PM2) | 25 |
|  | ANK2 | Сhr4:113264907, rs786205722,  NM_001148.6:c.1397C>T:p.**T466M** | VUS (BP1, PP2, PP3, PP4) | 27 |
| Patient 10 | PKP2 | Chr12:32878525,  NM_001005242.3:c.355delT:p.**Y119Mfs*23** | LP (PVS1, PM2) | - |
|  | PKP2 | Chr:32792645,  NM_001005242.3:c.2444A>G:p.**K815R** | VUS (PP3, PM2, BP1) | 33 |
| Patient 11 | PKP2 | Chr12:32878134,  NM_001005242.3:c.746G>C:p.**S249T** | LP (PM5, PM2, PP3, PP4) | 23 |
|  | PKP2 | Chr12:32877908,  NM_001005242.3:c.973dup:p.**A325Gfs*11** | LP (PVS1, PM2) | - |
| Patient 12 | DSP | Chr6:7583495, rs377035113,  NM_004415.4:c.6233T>C:p.**I2078T** | VUS (PM2, BP1) | 26 |
| Patient 13 | SCN5A | Chr3:38613781, rs45546039,  NM_198056.3:c.665G>A:p.**R222Q** | P (PS3, PP3, PM1, PM5, PM2) | 23 |
|  | ANK2 | Chr4:113355777, rs781642042,  NM_001148.6:c.7159G>C:p.**A2387P** | VUS (BP1, PM2, PP3, PP4) | 11 |
